# Associations Between Numeracy, Mathematics Anxiety, Perceived Teaching Quality and Medication Calculation Competence Among Undergraduate Nursing Students: A Cross‐Sectional Study

**DOI:** 10.1002/nop2.70679

**Published:** 2026-07-15

**Authors:** Tamer Al‐Ghraiybah, Zainab Zahr, Sanmei Chen, Chiara Torrelli, Jacqueline Pich, Leila Gholizadeh

**Affiliations:** ^1^ School of Nursing and Midwifery, Faculty of Health University of Technology Sydney Ultimo New South Wales Australia; ^2^ Global Health Nursing, Graduate School of Biomedical and Health Sciences Hiroshima University Hiroshima Japan

**Keywords:** medication calculation competence, medication safety, numeracy, nursing education, teaching quality

## Abstract

**Aim:**

To explore the associations of basic numeracy, mathematics anxiety and perceived quality of mathematics teaching with medication calculation competence among undergraduate nursing students.

**Design:**

A cross‐sectional survey design.

**Methods:**

A cross‐sectional survey among 111 undergraduate nursing students who had completed at least one semester of study was conducted at a metropolitan university in Sydney, Australia. Participants completed an anonymous online survey comprising the Drug Calculation Assessment Tool (DCAT), the Short Mathematics Anxiety Rating Scale, the Lipkus Numeracy Scale, and items assessing the perceived quality of mathematics teaching. Fractional logistic regression with robust standard errors was used to model medication‐calculation competence.

**Results:**

Higher basic numeracy was associated with better medication calculation competence (adjusted average marginal effects [AME]: 0.033, 95% confidence interval [CI]: 0.002–0.063, *p* = 0.037). Compared with students who rated teaching as poor or fair, those who rated it good or higher had significantly higher medication‐calculation competence (adjusted AME [95% CI]: 0.12 [0.036–0.198], *p* = 0.005). Mathematics anxiety was significantly associated with medication calculation competence in unadjusted analysis (AME [95% CI]: −0.027 [−0.053 to −0.001], *p* = 0.041), but this association was attenuated and became non‐significant after adjustment for numeracy (*p* = 0.363).

**Conclusion:**

Basic numeracy and perceived quality of mathematics teaching were the modifiable factors most consistently associated with medication calculation competence, whereas the association with mathematics anxiety was attenuated after adjustment for numeracy. These exploratory associations require confirmation in larger, multi‐site studies before firm conclusions can be drawn.

**Implications for the Profession and/or Patient Care:**

Strengthening foundational numeracy and the quality of mathematics instruction in undergraduate nursing curricula may support the development of safe medication‐calculation skills. Early numeracy screening and targeted, curriculum‐embedded support warrant evaluation as strategies to enhance graduates' clinical readiness and medication safety.

**Reporting Method:**

The study was reported in accordance with the Strengthening the Reporting of Observational Studies in Epidemiology (STROBE) checklist for cross‐sectional studies.

**Patient or Public Contribution:**

No Patient or Public Contribution.

## 1 Introduction

The administration of medications is a foundational, high‐accountability dimension of registered nursing practice and is among the most frequent and high‐risk clinical responsibilities undertaken in acute and community settings. The safe administration of medicines requires integrating clinical knowledge, critical thinking, pharmacological understanding and technical precision (Rohde and Domm 2018). Within this broader construct, medication competence is fundamental to ensuring patient safety and preventing avoidable harm (Donaldson et al. 2017). Medication competence encompasses multiple sequential processes, including accurate interpretation of medication orders, appropriate dose calculation, preparation, administration, monitoring and documentation (Thelen 2022). Among these processes, the ability to perform accurate medication calculations is particularly critical, as it directly determines the dose delivered to the patient and therefore the likelihood of either a therapeutic benefit or adverse outcome (Stake‐Nilsson et al. 2022). A large European study identified that medication calculation errors constitute a substantial proportion of medication administration errors, with estimates suggesting that approximately 38% of medication errors involve incorrect dosing calculations (Mulac et al. 2021). These findings underscore that medication calculation competency is not a peripheral skill but a central determinant of safe medication practice. As registered nurses are responsible for the majority of medication administration in patient settings, including dose preparation, calculation verification, and infusion rate adjustment, the development of accurate calculation skills during undergraduate education is essential to ensure patient safety upon entry to practice.

Medication calculation errors commonly arise from two interrelated sources. First, they may reflect deficits in fundamental numeracy skills, including difficulties with basic arithmetic operations such as division, multiplication, ratios and unit conversions (Williams and Davis 2016). The second arises from errors that may stem from challenges in conceptually interpreting medication problems, whereby nurses or nursing students are unable to accurately extract and organise relevant clinical information to formulate the appropriate mathematical expression (Vaismoradi et al. 2014). This conceptual dimension requires more than procedural computation; it involves understanding dose response principles, recognising units of measurement, identifying the required dose relative to the available stock strength, and translating clinical prescriptions into structured calculations (McMullan et al. 2010).

Nursing students are progressively integrated into medication administration throughout their undergraduate education, moving from supervised practice to increasingly independent roles as they advance through clinical placements. Competency in medication calculations is therefore not only an academic requirement but a prerequisite for safe participation in clinical care. Despite this, evidence consistently demonstrates that a substantial proportion of nursing students struggle with medication calculation tasks (Elonen et al. 2022; Minty‐Walker et al. 2024). International research has reported that more than half of nursing students fail to meet numeracy and medication calculation competency assessments at least once during their training, indicating that both computational and conceptual weaknesses remain prevalent in preregistration education (Stake‐Nilsson et al. 2022). These weaknesses are not confined to the undergraduate years: McMullan et al. (2010) found that registered nurses, as well as nursing students, performed poorly on medication calculation assessments, with frequent errors in basic arithmetic and unit conversion, and analyses of hospital incident reports have identified dose miscalculations, incorrect unit conversions, and tenfold dosing errors among practising nurses as recurrent contributors to severe and fatal medication errors (Mulac et al. 2021).

Informed by Bandura's (1986) Social Cognitive Theory, medication calculation competence can be conceptualised as the product of reciprocal interactions among personal factors, such as numeracy ability and mathematics anxiety; environmental factors, such as the quality of instruction; and behavioural factors, including engagement with practice opportunities. This framework guided the selection of the variables examined in this study and structures the review that follows.

Given the computational and conceptual difficulties identified in both student and practising nurse populations, attention has increasingly turned to factors that may underlie them. A growing body of literature has attributed, at least in part, problems in medication calculation performance to mathematics anxiety (Abdul‐Mumin et al. 2025; McKenna et al. 2022; Williams and Davis 2016). Mathematics anxiety is characterised by feelings of tension, apprehension, and avoidance that arise when individuals are confronted with numerical tasks, potentially interfering with working memory, concentration, and overall calculation accuracy (Alexander and Martray 1989; McKenna et al. 2022). Mathematics anxiety is commonly reported among nursing students, many of whom report fear and discomfort during medication calculation assessments (McKenna et al. 2022). Evidence across health disciplines links mathematics anxiety to impaired working memory, reduced concentration, and lower self‐efficacy, all of which may negatively affect calculation performance (Abdul‐Mumin et al. 2025; Khasawneh et al. 2021; McKenna et al. 2022; Williams and Davis 2016). Negative experiences with mathematics during earlier schooling often persist into tertiary education and re‐emerge during high‐stakes numeracy assessments (Di Martino and Gregorio 2019). Furthermore, declining enjoyment and confidence in mathematics across the school years may limit the development of the sustained competence required for accurate clinical calculation (Di Martino and Gregorio 2019).

The independent role of mathematics anxiety in medication calculation competence remains unclear. It is uncertain whether anxiety directly impairs performance or whether its apparent effect reflects underlying numeracy deficits (Skagerlund et al. 2019). Students with weaker foundational skills are more likely to experience anxiety, suggesting that skill deficits may be the more immediate determinant of poor performance (McKenna et al. 2022). Few studies have examined numeracy and mathematics anxiety simultaneously within the same cohort, limiting clarity regarding their relative contributions (McKenna et al. 2022).

A further consideration concerns the quality of mathematics instruction within nursing programs. A national survey of 170 Australian nurse academics reported that nearly half experienced anxiety or low confidence when teaching medication calculations, and approximately 90% had received no formal preparation in numeracy pedagogy (Minty‐Walker et al. 2023). Many education leaders identified primarily as clinicians rather than numeracy educators, indicating that while they could teach procedural formulae, they lacked the expertise to remediate foundational mathematical deficits (Minty‐Walker et al. 2023). Consistent with this concern, a systematic review of digital and pedagogical approaches to medication dosage calculation found that web‐based courses were the most common intervention yet demonstrated limited impact on skill development, and no single teaching method showed consistent effectiveness (Stake‐Nilsson et al. 2022). Although strategies emphasising conceptual understanding, such as proportional reasoning and ratio‐based logic, have been advocated as alternatives to rote formula memorisation, their integration into nursing curricula remains limited (Dubovi et al. 2018). Within Social Cognitive Theory, the instructional environment shapes learning through observational modelling, guided practice and feedback (Bandura 1986); students' perceptions of teaching quality therefore represent a proximal indicator of this environmental influence, yet one that has rarely been quantified in relation to competence in medication calculation.

These instructional limitations are further intensified by structural inconsistencies within Australian nursing education. Evidence demonstrates substantial variation in how numeracy is assessed across universities, with no national benchmark or standardised framework to guide practice (Minty‐Walker et al. 2024). Only a small proportion of programs require secondary school mathematics as an entry prerequisite, and accreditation standards provide no explicit direction regarding numeracy content, pedagogy, or assessment within undergraduate curricula (Minty‐Walker et al. 2024). Consequently, many educators assume that students commence their studies with adequate mathematical preparation, an assumption that is often not met. In numerous programs, there is no dedicated mathematics instruction, and students are expected to acquire medication‐calculation competence without a structured foundation in the mathematical principles underpinning safe medication practice.

These gaps are consequential because competence in medication calculation is inherently multidimensional. As outlined above, Social Cognitive Theory (Bandura 1986) frames medication calculation competence as the product of reciprocally interacting personal, environmental and behavioural determinants. Despite this theoretical framing, few Australian studies have examined cognitive, psychological, and instructional variables simultaneously within the same cohort to determine their relative contributions to medication calculation performance. Identifying which factors most strongly predict competence is critical for directing educational investment toward the domains most likely to improve medication safety outcomes.

This study aimed to address this gap by examining the associations between mathematics anxiety, basic numeracy, perceived quality of mathematics teaching, mathematics recency, and medication calculation competence among undergraduate nursing students at a metropolitan Australian university. It was hypothesised that: (1) higher basic numeracy would be associated with greater medication calculation competence; (2) higher mathematics anxiety would be associated with lower competence; (3) higher perceived quality of mathematics teaching would be associated with greater competence; and (4) the association between mathematics anxiety and competence would attenuate after adjustment for numeracy.

## 2 Methods

### 2.1 Study Design

A cross‐sectional survey design was used to examine the associations between mathematics anxiety, basic numeracy, mathematics recency, perceived quality of mathematics teaching, and medication calculation competence among undergraduate nursing students. The study is reported in accordance with the Strengthening the Reporting of Observational Studies in Epidemiology (STROBE) guidelines (Von Elm et al. 2007). A completed STROBE checklist for cross‐sectional studies is provided as Supporting Information (see Data S1).

### 2.2 Setting and Participants

The study was conducted at a metropolitan university in Sydney, New South Wales, Australia, offering a 3‐year Bachelor of Nursing program. All undergraduate nursing students who had completed at least one semester of study were eligible to participate.

### 2.3 Data Collection

Data were collected between October and December 2025 using an anonymous online survey administered via the Qualtrics platform. This period was selected to coincide with a lower assessment load, thereby minimising potential burden on participants. Institutional ethics approval was obtained prior to the commencement of data collection.

Study materials, including the participant information sheet and survey link, were disseminated by year coordinators and professional staff to reduce the risk of perceived coercion by research team members. Initial recruitment involved a general email invitation to all eligible students, followed by a personalised reminder email to increase the response rate. Participation was entirely voluntary and anonymous. The survey took approximately 20 min to complete. Qualtrics settings were configured to prevent multiple submissions, and any duplicate responses identified during data cleaning were removed.

### 2.4 Instruments

The survey comprised five sections: demographic characteristics, medication calculation competence, mathematics anxiety, basic numeracy, and academic mathematics learning and support.

#### 2.4.1 Demographic Characteristics

Participants reported their age, gender, year of study (Year 1, 2 or 3), and domestic or international enrolment status.

#### 2.4.2 Medication Calculation Competence

Medication calculation competence was assessed using the Drug Calculation Assessment Tool (DCAT), a 15‐item instrument developed for this study. The DCAT comprises four sections: basic medication calculations (4 items), intravenous flow rate calculations (4 items), paediatric and weight‐based dosing (3 items), and unit conversions (4 items). The tool was tailored to match the mathematical content of students' year of study: Year 1 students completed basic medication calculations and unit conversions, whilst Year 2 and Year 3 students completed all sections. The DCAT yields a percentage score ranging from 0 to 100, with higher scores indicating greater competence. The test was reviewed by nursing academics with expertise in medication calculations for face validity, with feedback and corrections incorporated. In the present study, the DCAT demonstrated excellent internal consistency (KR‐20 = 0.97). This very high internal consistency should be interpreted with caution: it may reflect redundancy or homogeneity among items and is likely to have been inflated by the pronounced ceiling effect in the score distribution. As the DCAT is newly developed and has been evaluated only for face validity, further psychometric evaluation, including analyses of item difficulty, item discrimination and dimensionality, is required before wider adoption. The Drug Calculation Assessment Tool, developed for this study, is provided as Supporting Information (Data S2).

#### 2.4.3 Mathematics Anxiety

Mathematics anxiety was measured using the Short Mathematics Anxiety Rating Scale (Alexander and Martray 1989), a 25‐item instrument comprising three subscales: mathematics test anxiety (15 items), numerical task anxiety (5 items), and mathematics course anxiety (5 items). Respondents rated each item on a five‐point Likert scale ranging from 1 (‘not at all’) to 5 (‘very much’), yielding a total score of 25–125, with higher scores indicating greater anxiety. In the present study, Cronbach's alpha for the total scale was 0.97, indicating excellent internal consistency.

#### 2.4.4 Numeracy Ability

Basic numeracy ability was assessed using the Lipkus Numeracy Scale, an 11‐item measure of fundamental mathematical competence (Lipkus et al. 2001). Each item was scored as correct or incorrect, yielding a total score of 0 to 11. In the present study, the Lipkus Numeracy Scale demonstrated good internal consistency (KR‐20 = 0.83).

#### 2.4.5 Mathematics Recency

Mathematics recency was derived from participants' self‐reported last year of formal mathematics study and dichotomised as Year 12 or later versus Year 10 or earlier for analysis.

#### 2.4.6 Quality of Mathematics Teaching

Perceived quality of mathematics teaching was assessed using a single investigator‐developed item (‘How would you rate the quality of math teaching you received during your nursing studies?’) rated on a five‐point scale from ‘poor’ to ‘excellent’ and dichotomised as higher quality (good/very good/excellent) versus poor/fair for regression modelling. A single global item was used because no validated instrument measuring perceived quality of mathematics teaching in nursing education could be identified, and to minimise respondent burden within an already lengthy survey. Single‐item global ratings of teaching quality have demonstrated acceptable concurrent validity relative to multi‐item instruments in health professional education (Williams et al. 2002); nonetheless, a single item cannot capture the multidimensional nature of instructional quality, which is acknowledged as a limitation.

### 2.5 Sample Size

An a priori sample size calculation was conducted using G*Power 3.1 (Faul et al. 2009). Assuming a small‐to‐medium effect size (*f* ^2^ = 0.05), a significance level of *α* = 0.05, and statistical power of 0.80, a minimum of 293 participants was required. To account for an anticipated 20% rate of incomplete responses, the target was adjusted upward to approximately 360. All eligible students (*n* = 1600) were invited to participate, yielding 111 completed responses (response rate 6.9%). The lower‐than‐anticipated response rate likely reflects the voluntary, anonymous nature of the survey, its distribution during the clinical placement period, and known challenges to online survey recruitment among undergraduate students (Shiyab et al. 2023; Vadeboncoeur et al. 2018). The achieved sample therefore represented approximately 38% of the required 293 participants, and the analytic sample for the adjusted model was further reduced to 97 by missing data. The study was consequently underpowered to detect small effects, and the adjusted estimates should be regarded as exploratory, with wider confidence intervals and reduced stability than originally planned. The implications of the sample size for the interpretation of findings are addressed in the limitations section.

### 2.6 Data Analysis

Data were cleaned in Microsoft Excel and analysed using Stata/MP 19.5 (StataCorp, College Station, TX, USA). Variables were checked for duplicates and invalid ranges, and categorical variables were verified against their value labels. Descriptive statistics were employed to summarise sample characteristics: continuous variables were reported as means and standard deviations, and categorical variables as frequencies and percentages. Cases with missing data on any analytic variable were excluded from the relevant analysis (complete‐case analysis), reducing the sample to 97 for the adjusted regression model.

Bivariate associations between medication calculation competence, mathematics anxiety, and basic numeracy ability were examined using Spearman's rank correlation coefficients. Variance inflation factors were used to assess multicollinearity among independent variables.

Medication calculation competence, measured as a percentage score (0–100), exhibited a pronounced ceiling effect. Accordingly, the primary analysis employed fractional logistic regression fitted to the proportion outcome, with robust standard errors. This approach is appropriate for outcomes bounded within [0, 1] and avoids predictions outside the feasible range, consistent with methodological recommendations for proportion outcomes (Papke and Wooldridge 1996). Results were reported as average marginal effects (AMEs) scaled to percentage points. Unadjusted models were estimated for each independent variable, followed by an adjusted multivariable model including the prespecified covariates age and year of study.

## 3 Results

A total of 111 nursing students participated (Table 1). The sample had a mean age of 23.8 years (SD 6.5) and was predominantly female (82.9%). Students were distributed across Year 1 (35.1%), Year 2 (44.1%), and Year 3 (20.7%), with 90.1% of students being domestic. Academic performance clustered toward higher achievement, with 61.3% achieving Distinction or higher. Self‐rated confidence in medication calculations was generally high, with 72% reporting moderately to very confident.

**TABLE 1:** Participant characteristics and key study variables (*N* = 111).

Variable	Mean (SD)/*N* (%)	Range
Age, years^a^	23.8 (6.5)	17–51
Sex		
Female	92 (82.9%)	
Male	16 (14.4%)	
Non‐binary	3 (2.7%)	
Grade		
High distinction	14 (12.6%)	
Distinction	54 (48.7%)	
Credit	28 (25.2%)	
Pass	14 (12.6%)	
Failed	1 (0.9%)	
Study year		
Year 1	39 (35.1%)	
Year 2	49 (44.1%)	
Year 3	23 (20.7%)	
Mathematics recency		
Before Year 12	35 (31.5%)	
Year 12 (or equivalent)	76 (68.5%)	
Nationality		
Domestic student	100 (90.1%)	
International student	11 (9.9%)	
Confidence in performing medication calculations		
Not confident	3 (2.7%)	
Slightly confident	15 (13.5%)	
Moderately confident	34 (30.6%)	
Very confident	46 (41.4%)	
Extremely confident	13 (11.7%)	
Perceived quality of maths teaching		
High	63 (56.8%)	
Poor/fair	48 (43.2)	
Short Mathematics Anxiety Rating Scale (sMARS), points	68.3 (23.9)	25–123
Maths Test Anxiety subscale (15 items), points	36.2 (13.3)	15–74
Numerical Task Anxiety subscale (5 items), points	16.8 (6.1)	5–25
Maths course Anxiety subscale (5 items), points	15.4 (5.5)	5–25
Basic numerical ability, points	9.8 (2.1)	1–11
High score (9–11)	90 (81.1%)	
Low score (0–8)	21 (18.9%)	
Medication competency, points	82.0 (23.0)	0–100
Pass	56 (50.5%)	
Fail	55 (49.5%)	

*Note:* Grade refers to participants' self‐reported most recent overall academic grade in the Bachelor of Nursing program, classified according to standard Australian university grading bands: high distinction (85%–100%), distinction (75%–84%), credit (65%–74%), pass (50%–64%) and fail (< 50%).

Abbreviation: SD, standard deviation.

^a^ Missing data = 3.

Most participants (71.4%) reported that their most recent formal mathematics study was Year 12 or later. Regarding perceived quality of mathematics teaching, 63 students (56.8%) rated teaching as good or above, while 48 (43.2%) rated it as poor or fair. The Mathematics Anxiety Scale yielded a mean total score of 68.3 (SD 23.9). Numeracy performance was high (mean 9.8, SD 2.1). Medication calculation competence averaged 82.0 (SD 23.0); 56 students (50.5%) met the pass criterion (100%), with scores clustering toward the upper end of the distribution.

Spearman correlations revealed that medication calculation competence was strongly and positively associated with numeracy (*ρ* = 0.66, *p* < 0.001). Medication calculation competence showed a weak negative correlation with mathematics anxiety, but this relationship was not statistically significant (*ρ* = −0.15, *p* = 0.113). Numeracy and mathematics anxiety also showed a weak inverse association that was not statistically significant (*ρ* = −0.16, *p* = 0.091). Variance inflation factors (VIF < 1.15) confirmed that there was no problematic multicollinearity among the independent variables.

In unadjusted analyses, higher numeracy was strongly associated with better medication calculation competence (AME 0.064, 95% CI 0.039–0.088, *p* < 0.001), corresponding to a 6.4 percentage‐point increase per one‐point increase in numeracy score (Table 2). Perceived teaching quality showed a substantial association: students reporting higher quality demonstrated 23.4% points higher competence than those reporting poor/fair quality (AME 0.234, 95% CI 0.135–0.333, *p* < 0.001). A higher level of mathematics anxiety was associated with lower competence, with each 10‐point increase corresponding to a 2.70 percentage‐point reduction (AME −0.027, 95% CI −0.053 to −0.001, *p* = 0.041). Mathematics recency was not significantly associated with competence (*p* = 0.174). In the adjusted model, numeracy and perceived teaching quality remained independently associated with competence (Table 2). Each one‐point increase in numeracy corresponded to a 3.3 percentage‐point increase (AME 0.033, 95% CI 0.002–0.063, *p* = 0.037), while high perceived teaching quality was associated with a 12 percentage‐point increase in competence, compared to poor/fair quality (AME 0.12, 95% CI 0.036–0.198, *p* = 0.005). Mathematics anxiety and mathematics recency showed no significant independent associations (both *p* > 0.05).

**TABLE 2:** Fractional logistic regression models predicting medication calculation competence.

	AME^a^ (d*y*/d*x*)	95% CI	*p*
Unadjusted models (*n* = 111)			
Maths anxiety (per 10‐point increase)	−0.027	−0.053 to −0.001	0.041
Numeracy (per 1‐point increase)	0.064	0.039 to 0.088	< 0.001
Perceived quality of maths teaching (Higher vs. Poor/Fair)	0.234	0.135 to 0.333	< 0.001
Mathematics recency (Year 12 vs. Year 10)	0.120	−0.050 to 0.300	0.174
Adjusted model (*n* = 97)^b^			
Maths anxiety (MARS10) (per 10‐point increase)	−0.01	−0.03 to 0.01	0.363
Numeracy (per 1‐point increase)	0.033	0.002 to 0.063	0.037
Perceived quality of maths teaching (Higher vs. Poor/Fair)	0.12	0.036 to 0.198	0.005
Mathematics recency (Year 12 vs. Year 10)	0.01	−0.10 to 0.12	0.876

Abbreviations: AME, average marginal effect; CI, confidence interval.

^a^ Multiply AMEs by 100 to express effects in percentage points on the 0–100 medication Cal scale (e.g., an AME of 0.117 ≈11.7 percentage points).

^b^ Adjusted model included all independent variables, including math anxiety (continuous variable), numeracy (continuous variable), perceived quality of maths teaching (high quality vs. poor/fair quality), mathematics recency (year 12 vs. year 10), and confound factors including age (continuous variable) and year of study (Year 1 vs. Year 2–3).

## 4 Discussion

This study examined the associations between mathematics anxiety, basic numeracy, perceived quality of mathematics teaching, mathematics recency and medication calculation competence among undergraduate nursing students at a metropolitan Australian university. Three principal findings emerged: basic numeracy was the strongest and most consistent correlate of medication calculation competence; perceived quality of mathematics teaching was independently associated with competence; and the association between mathematics anxiety and competence attenuated after adjustment for numeracy. These findings are interpreted below through the lens of Social Cognitive Theory (Bandura 1986), which conceptualises competence as arising from reciprocal interactions among personal, environmental and behavioural factors.

Across the primary fractional logistic regression analyses, basic numeracy emerged as the most robust correlate of medication calculation competence. Spearman's correlation analysis confirmed a strong positive association between numeracy and medication‐calculation competence (*ρ* = 0.66, *p* < 0.001). In the adjusted model, each one‐point increase in numeracy corresponded to a 3.27 percentage‐point increase in competence (AME 0.033, 95% CI 0.002–0.063, *p* = 0.037). This finding is consistent with McMullan et al. (2010), who reported that foundational arithmetic skills were a key determinant of medication calculation accuracy among both nursing students and registered nurses, with Elonen et al. (2022), who stated that European nursing graduates struggling with multi‐step medication calculations frequently demonstrated underlying deficits in basic mathematical operations, and with Stake‐Nilsson et al. (2022), who concluded in their systematic review of digital technologies for medication dosage calculation that no technological intervention can compensate for deficiencies in students' basic mathematical competence, noting that calculators and web‐based tools are ineffective when foundational knowledge is lacking. The present findings extend this evidence by quantifying the strength of the numeracy–competence association in an Australian cohort and by demonstrating that this relationship persists after controlling for anxiety, teaching quality and relevant demographic variables.

The clinical importance of these findings warrants emphasis. Westbrook et al. (2015) demonstrated that over 27% of medication administrations at two major Australian hospitals contained at least one error, with the majority of clinically important prescribing errors going undetected. Students who graduate with weak numeracy foundations may therefore be entering clinical environments where existing safeguards do not reliably detect calculation errors. In Australia, medication errors remain the second most‐reported type of preventable incident in hospitals (Semple and Roughhead 2009), and evidence suggests that a substantial proportion of errors, including dosing errors, go unreported or undetected through routine incident reporting systems (Westbrook et al. 2020). Within the framework of Bandura (1986) Social Cognitive Theory, numeracy functions as a core personal factor that directly influences performance attainments and self‐efficacy beliefs. Students with stronger mathematical competence are better equipped to engage successfully with medication calculation tasks, reinforcing their confidence through mastery experiences. Conversely, those with weaker foundations may experience repeated difficulty, diminish self‐efficacy and increase avoidance behaviours. This is particularly concerning given Minty‐Walker et al. (2023) finding that nurse education leaders assumed students would arrive at university with adequate numeracy, only to discover this was frequently not the case. The strength and consistency of the numeracy–competence relationship in the present study reinforce the need for early identification and structured remediation of numeracy deficits, rather than treating medication calculation competence as a skill students will acquire incidentally through clinical exposure.

A notable feature of the context in which this study was conducted is the absence of dedicated mathematics teaching within the nursing curriculum and the lack of a prerequisite mathematics requirement for entry into the programme. Students are therefore expected to develop and apply competence in medication calculation without a structured foundation in the mathematical principles that underpin it. This curricular gap may partly explain the wide variation in numeracy scores observed in this cohort and raises important questions about the assumptions embedded in current programme design. Many Australian nursing programmes share this characteristic (Minty‐Walker et al. 2024). Yet few studies have examined the consequences of entering nursing education without formal mathematics screening or ongoing numeracy instruction. Future research should therefore investigate the feasibility and effectiveness of embedding structured numeracy teaching within the nursing curriculum.

Of particular interest are teaching strategies grounded in logical reasoning, such as ratio and proportion frameworks, which emphasise conceptual understanding of the relationships among ordered, supplied, and administered doses rather than reliance on rote memorisation of formulae. Evidence from mathematics education research suggests that proportional reasoning is foundational to multiplicative thinking and that explicit instruction in this area can improve performance on applied calculation tasks (Dubovi et al. 2018; Wright 2011). Integrating such approaches into nursing curricula through dedicated tutorials, worked examples using authentic clinical scenarios, and scaffolded practice opportunities may offer a more sustainable pathway to competence than isolated revision sessions or self‐directed online modules (Dubovi et al. 2018; Mariani et al. 2017; Rainboth and DeMasi 2006).

In the primary adjusted model, students who rated the quality of mathematics teaching as good or above demonstrated 11.71 percentage points higher medication calculation competence compared with those who rated teaching as poor or fair (AME 0.117, 95% CI 0.036–0.198, *p* = 0.005). This finding suggests that instructional quality, as perceived by students, contributes meaningfully to calculation performance beyond what can be accounted for by numeracy ability alone.

This finding offers empirical support for previously qualitative concerns regarding numeracy instruction in nursing education. The majority of evidence indicates that many nurse educators identify primarily as clinicians, lack formal preparation in numeracy pedagogy, and report low confidence when teaching medication calculations (Minty‐Walker et al. 2023). The present study suggests that such educator‐level limitations have measurable implications for student performance. This is further situated within a fragmented national context characterised by substantial variation in numeracy assessment and the absence of standardised benchmarks (Minty‐Walker et al. 2024). In such an environment, variability in perceived teaching quality is likely to influence competence independently.

Evidence indicates that web‐based courses are the most common digital intervention for medication dosage calculation, yet have limited impact on skill development, with no single technological approach demonstrating consistent effectiveness (Stake‐Nilsson et al. 2022). In contrast, interdisciplinary collaboration that scaffolds quantitative reasoning across disciplines has been associated with reduced mathematics anxiety and improved engagement (Post et al. 2022). These findings suggest that effectiveness depends less on delivery format and more on pedagogical expertise and collaborative design. From a Social Cognitive Theory perspective (Bandura 1986), the teaching environment is a key contextual influence on guided practice, feedback, and the development of competence and self‐efficacy. Investment in educator capability and evidence‐based instructional design may therefore improve student outcomes.

These findings highlight the need for research evaluating interventions that strengthen numeracy instruction within nursing programmes. Studies assessing structured professional development for nurse educators, including collaborative models, are required to determine whether improved teaching quality leads to measurable gains in student competence (Post et al. 2022). Further intervention research should examine whether prerequisite numeracy screening with targeted remediation enhances calculation performance and confidence across the degree.

Mathematics anxiety was significantly associated with lower medication calculation competence in unadjusted analyses (AME −0.027, *p* = 0.041), consistent with a body of literature identifying anxiety as a barrier to numeracy performance in health professions students. Abdul‐Mumin et al. (2025) reported that nursing students frequently experience fear and discomfort during medication calculation assessments, while Hossain et al. (2025) and Khasawneh et al. (2021) demonstrated links between mathematics anxiety, impaired working memory, and reduced self‐efficacy. McKenna et al. (2022) noted that negative experiences with mathematics during schooling often persist into tertiary education, resurfacing during high‐stakes numeracy assessments. These findings collectively informed the expectation that anxiety would function as an independent factor in the present study.

However, the association was attenuated and no longer statistically significant after adjusting for numeracy and other covariates (*p* = 0.363). This attenuation suggests that much of what has been attributed to mathematics anxiety in the existing literature may operate through, or be confounded with, underlying numeracy deficits. Students with weaker foundational skills are more likely to experience anxiety, and it may be this skill deficit—rather than the affective response itself—that most directly impairs calculation performance.

This finding has important implications for how poor medication calculation performance is conceptualised within the profession. Evidence suggests that declining enjoyment of mathematics and limited curricular scaffolding contribute to anxiety, particularly when quantitative reasoning is taught as a high‐stakes, isolated subject rather than embedded within clinical contexts (Post et al. 2022; Russo et al. 2023). The present results indicate that approaches focused primarily on anxiety reduction may target a downstream effect rather than the underlying deficit. Consistent with Social Cognitive Theory (Bandura 1986), affective states influence performance largely through self‐efficacy and prior mastery experiences rather than as independent causal agents. Strengthening numeracy competence may therefore represent the more effective pathway to improving performance and indirectly reducing anxiety. Nonetheless, the observed unadjusted association suggests that emotional factors should not be ignored, and curricula that reduce threat and foster supportive learning environments may complement skill‐based strategies.

Future research should examine the anxiety–competence relationship using longitudinal designs that track trajectories in numeracy, anxiety, and competence from enrolment through to graduation and into early clinical practice. Such designs would provide a more comprehensive understanding of how these factors interact over time and clarify whether anxiety exerts its influence primarily through its association with numeracy or retains an independent effect at developmental stages or assessment thresholds. This research would directly inform curriculum reform and contribute to the development of national standards for numeracy preparation in undergraduate nursing education.

### 4.1 Limitations

Several limitations warrant consideration. First, the achieved sample (*n* = 111) represented only approximately 38% of the a priori target of 293, with the analytic sample further reduced to 97 in the adjusted model; the study was therefore underpowered to detect small effects, and the precision and stability of the adjusted estimates should be interpreted with caution. Second, the response rate was low (6.9%), raising a substantial risk of self‐selection bias: students with greater mathematical confidence may have been more willing to participate, which would inflate competence and numeracy estimates and may have attenuated the observed associations through restriction of range. Because the survey was anonymous, demographic comparisons between respondents and non‐respondents were not available, although the clustering of self‐reported grades toward Distinction or higher (61.3%) suggests that higher‐achieving students may have been over‐represented. Third, the perceived quality of mathematics teaching was self‐reported using a single retrospective item, which may introduce recall and satisfaction bias and may not adequately capture the complexity of instructional experiences. Although single‐item global ratings have shown concurrent validity with multi‐item instruments (Williams et al. 2002), findings related to this variable should nonetheless be interpreted with caution, and the development and validation of a multi‐item measure of perceived quality of mathematics teaching in nursing education is recommended for future research. Fourth, the DCAT was newly developed and evaluated only for face validity; its very high internal consistency (KR‐20 = 0.97) may partly reflect item redundancy and is likely to have been inflated by the pronounced ceiling effect, and further psychometric evaluation is required. Fifth, the cross‐sectional design precludes causal inference regarding the relationships between numeracy, teaching quality and competence, and data collection during clinical placement may have influenced participation and responses. Finally, the study was conducted at a single metropolitan university with a predominantly female and domestic cohort, which may limit generalisability; however, as the programme shares key structural characteristics with many Australian nursing programmes, including the absence of a mathematics prerequisite for entry and the lack of dedicated numeracy instruction (Minty‐Walker et al. 2024), the findings are likely to be relevant to comparable programmes nationally, pending confirmation in multi‐site studies encompassing regional, rural and more culturally diverse cohorts.

## 5 Conclusion

This study demonstrates that foundational numeracy and the perceived quality of mathematics instruction are the modifiable factors most strongly associated with medication‐calculation competence among undergraduate nursing students. Mathematics anxiety showed an association at the bivariate level but did not appear to function as an independent determinant once numeracy was considered. The findings suggest that strengthening core numeracy skills and improving instructional quality may be more effective than focusing solely on reducing anxiety. Prioritising these domains within nursing curricula has the potential to enhance clinical readiness and support safer medication administration practice.

## Author Contributions

**Tamer Al‐Ghraiybah:** data curation, formal analysis, investigation, methodology, data interpretation, software, and writing original draft and review and editing; **Zainab Zahr:** conceptualization, data curation, investigation, methodology, data interpretation, software, project administration, resources, and writing original draft and review and editing; **Sanmei Chen:** methodology, data interpretation, and writing review and editing; **Chiara Torrelli:** data curation, investigation, and writing original draft and review and editing; **Jacqueline Pich:** methodology, data interpretation, and writing review and editing; **Leila Gholizadeh:** methodology, data interpretation, and writing original draft and review and editing. All authors read and approved the final version of the manuscript.

## Funding

The authors have nothing to report.

## Ethics Statement

Ethical approval was granted by the University of Technology Sydney Human Research Ethics Committee (HREC) (approval number: ETH25‐11296).

## Consent

Informed consent was implied by the voluntary completion and submission of the survey. All responses were anonymous, and no identifying information was collected.

## Conflicts of Interest

The authors declare no conflicts of interest.

## Supporting information


**Data S1:** Supporting Information.

**Data S2:** Supporting Information.

## Data Availability

The data that support the findings of this study are available from the corresponding author upon reasonable request.
