# Supplementary material for: Associations Between Numeracy, Mathematics Anxiety, Perceived Teaching Quality and Medication Calculation Competence Among Undergraduate Nursing Students: A Cross‐Sectional Study
Source: Nurs Open. 2026 Jul 15;13(7):e70679. doi: 10.1002/nop2.70679 (PMC13370204; doi:10.1002/nop2.70679)
Supplement: Supplementary file 1 — Data S1: Supporting Information. [file NOP2-13-e70679-s001.docx]

**STROBE Statement—Checklist of items that should be included in reports of *cross-sectional studies***

| **Topic** | **Item No** | **Recommendation** | **Page No** |
| --- | --- | --- | --- |
| **Title and abstract** | 1 | (*a*) Indicate the study’s design with a commonly used term in the title or the abstract | 1 |
|  |  | (*b*) Provide in the abstract an informative and balanced summary of what was done and what was found | 1–2 |
| **Introduction** | | | |
| **Background/rationale** | 2 | Explain the scientific background and rationale for the investigation being reported | 3–6 |
| **Objectives** | 3 | State specific objectives, including any prespecified hypotheses | 7–8 |
| **Methods** | | | |
| **Study design** | 4 | Present key elements of study design early in the paper | 8 |
| **Setting** | 5 | Describe the setting, locations, and relevant dates, including periods of recruitment, exposure, follow-up, and data collection | 8–9 |
| **Participants** | 6 | (*a*) Give the eligibility criteria, and the sources and methods of selection of participants | 8–9 |
| **Variables** | 7 | Clearly define all outcomes, exposures, predictors, potential confounders, and effect modifiers. Give diagnostic criteria, if applicable | 9–11 |
| **Data sources/ measurement** | 8* | *For each variable of interest, give sources of data and details of methods of assessment (measurement). Describe comparability of assessment methods if there is more than one group* | 9–11 |
| **Bias** | 9 | Describe any efforts to address potential sources of bias | 9, 20 |
| **Study size** | 10 | Explain how the study size was arrived at | 11 |
| **Quantitative variables** | 11 | Explain how quantitative variables were handled in the analyses. If applicable, describe which groupings were chosen and why | 10–12 |
| **Statistical methods** | 12 | (*a*) Describe all statistical methods, including those used to control for confounding | 12 |
|  |  | (*b*) Describe any methods used to examine subgroups and interactions | NA |
|  |  | (*c*) Explain how missing data were addressed | 12 |
|  |  | (*d*) If applicable, describe analytical methods taking account of sampling strategy | NA |
|  |  | (*e*) Describe any sensitivity analyses | NA |
| **Results** | | | |
| **Participants** | 13* | (a) Report numbers of individuals at each stage of study—eg numbers potentially eligible, examined for eligibility, confirmed eligible, included in the study, completing follow-up, and analysed | 11, 13 |
|  |  | (b) Give reasons for non-participation at each stage | NA |
|  |  | (c) Consider use of a flow diagram | NA |
| **Descriptive data** | 14* | (a) Give characteristics of study participants (eg demographic, clinical, social) and information on exposures and potential confounders | 13, 25 |
|  |  | (b) Indicate number of participants with missing data for each variable of interest | 13, 25 |
| **Outcome data** | 15* | Report numbers of outcome events or summary measures | 13 |
| **Main results** | 16 | (*a*) Give unadjusted estimates and, if applicable, confounder-adjusted estimates and their precision (eg, 95% confidence interval). Make clear which confounders were adjusted for and why they were included | 13–14, 26 |
|  |  | (*b*) Report category boundaries when continuous variables were categorized | 10–11, 25 |
|  |  | (*c*) If relevant, consider translating estimates of relative risk into absolute risk for a meaningful time period | NA |
| **Other analyses** | 17 | Report other analyses done—eg analyses of subgroups and interactions, and sensitivity analyses | NA |
| **Discussion** | | | |
| **Key results** | 18 | Summarise key results with reference to study objectives | 14 |
| **Limitations** | 19 | Discuss limitations of the study, taking into account sources of potential bias or imprecision. Discuss both direction and magnitude of any potential bias | 20 |
| **Interpretation** | 20 | Give a cautious overall interpretation of results considering objectives, limitations, multiplicity of analyses, results from similar studies, and other relevant evidence | 14–21 |
| **Generalisability** | 21 | Discuss the generalisability (external validity) of the study results | 21 |
| **Other information** | | | |
| **Funding** | 22 | Give the source of funding and the role of the funders for the present study and, if applicable, for the original study on which the present article is based | Title Page |

**Give information separately for exposed and unexposed groups.*
