# Supplementary material for: Associations Between Numeracy, Mathematics Anxiety, Perceived Teaching Quality and Medication Calculation Competence Among Undergraduate Nursing Students: A Cross‐Sectional Study
Source: Nurs Open. 2026 Jul 15;13(7):e70679. doi: 10.1002/nop2.70679 (PMC13370204; doi:10.1002/nop2.70679)
Supplement: Supplementary file 2 — Data S2: Supporting Information. [file NOP2-13-e70679-s002.docx]

Section 4: Drug Calculation Ability Test (DCAT)

This section assesses your ability to perform basic drug calculations commonly encountered in nursing practice. The questions are designed to reflect real-world clinical scenarios involving medication dosages, conversions, and administration. Please attempt each question to the best of your ability—your responses will help identify areas of strength and opportunities for further support in nursing education.

| Part A:  Basic Dosage Calculations | Answer : |
| --- | --- |
| 1. Order: Amoxicillin 500 mg PO.  Supply: 250 mg tablets.   How many tablets? |  |
| 1. Order: Digoxin 0.25 mg PO.  Supply: 0.125 mg tablets.   How many tablets? |  |
| 1. Order: Furosemide 40 mg IV.   Supply: 10 mg/mL.  How many mL? |  |
| 1. Order: Morphine 6 mg IV.  Supply: 4 mg/mL.   How many mL? |  |
| Part B:  IV Flow Rate Calculations | Answer: |
| 1. Order: Normal Saline 1000 mL over 8 hours.  Tubing: 20 drops per min   What is the drip rate (per min)? |  |
| 1. Order: Vancomycin 1 g in 250 mL over 90 minutes.   Pump setting (mL/hr)? |  |
| 1. Order: Heparin 25,000 units in 500 mL at 12 mL/hr.   How many units/hr is the patient receiving? |  |
| 1. Order: Insulin drip 50 units in 100 mL at 5 mL/hr.   How many units/hr? |  |
| Part C:  Paediatric & Weight-Based Dosing | Answer: |
| 1. Order: Paracetamol 15 mg/kg for a child weighing 24 kg. Supply: 120 mg/5 mL How many mL? |  |
| 1. Order: Amoxicillin 30 mg/kg/day in 3 divided doses for a 15 kg child. Per-dose amount (mg)? |  |
| 1. Order: Gentamicin 5 mg/kg/day in 2 doses for a 70 kg adult. Per-dose amount (mg)? |  |
| Part D: Unit Conversions | Answer: |
| 1. Convert 0.5 g to mg. |  |
| 1. Convert 2500 mcg to mg. |  |
| 1. Convert 1.2 L to mL. |  |
| 1. Convert 0.04 mg to mcg. |  |
